# Smoking‐induced radiation laryngeal necrosis after definitive radiotherapy alone for T1a glottic squamous cell carcinoma: A case report

**DOI:** 10.1002/cnr2.1530

**Published:** 2021-08-15

**Authors:** Yoshiaki Takagawa, Sachiko Izumi, Minoru Aoki, Yuka Umeda, Kazuto Ochiai, Junko Kumada, Muneo Nakaya, Yuichiro Kadomatsu, Shingo Itagaki, Midori Kita

**Affiliations:** ^1^ Department of Radiation Oncology Southern Tohoku Proton Therapy Center Fukushima Japan; ^2^ Department of Radiology Tokyo Metropolitan Tama Medical Center Tokyo Japan; ^3^ Department of Otolaryngology – Head and Neck Surgery Tokyo Metropolitan Tama Medical Center Tokyo Japan; ^4^ Department of Pathology Tokyo Metropolitan Tama Medical Center Tokyo Japan

**Keywords:** glottic cancer, laryngeal necrosis, late toxicity, radiotherapy, smoking

## Abstract

**Background:**

We report the case of a patient with smoking‐induced radiation laryngeal necrosis (RLN) after undergoing definitive radiotherapy (RT) alone for T1a glottic squamous cell carcinoma.

**Case:**

The patient was a 63‐year‐old man who had a history of heavy smoking. He quit smoking when he was diagnosed with glottic squamous cell carcinoma. The RT dose was 63 Gy, delivered in 28 fractions with the three‐dimensional conventional RT technique for the larynx. After RT completion, the initial treatment response was complete response. He then underwent follow‐up examinations. At 13 months after RT, the patient resumed smoking. At 2 months after resuming smoking, he had severe sore throat and hoarseness. Laryngoscopy revealed a large tumor in the glottis. Surgical excision was performed, and the patient was histologically diagnosed with RLN, as late toxicity without cancer recurrence. At 3 weeks postoperatively, the patient had dyspnea, and laryngoscopy revealed total laryngeal paralysis. Thus, he underwent an emergent tracheostomy. The administration of steroids affected RLN, and laryngeal paralysis gradually improved.

**Conclusions:**

This case suggests that smoking may have the potential to induce RLN after RT. Moreover, continuing smoking cessation is significantly important for patients with glottic cancer who receive RT. Rather than leaving smoking cessation up to the patient, it would be necessary for clinicians to actively intervene to help patients continue their effort to quit smoking.

## 1 INTRODUCTION

Radiotherapy (RT) alone is the standard treatment for T1 glottic squamous cell carcinoma. RT alone demonstrates a 10‐year local control rate of >90% for T1 glottic squamous cell carcinoma.^1^, ^2^ Although the severe complication rate is low, radiation laryngeal necrosis (RLN) is a serious late toxicity of RT. In a previous report, the incidence rate of RLN was reported to be 2–3%.^3^, ^4^ Treating RLN is significantly difficult, and the incidence of total laryngectomy is high in patients with RLN. The main pathology of RLN is chondronecrosis. Smoking is significantly associated with the occurrence and recurrence of glottic squamous cell carcinoma.^5^, ^6^ Moreover, smoking causes vocal cord mucosal ischemia, which is also a risk factor for RLN. Here, we report a case of smoking‐induced RLN after definitive RT for T1a glottic squamous cell carcinoma.

## 2 CASE REPORT

**FIGURE 1:**
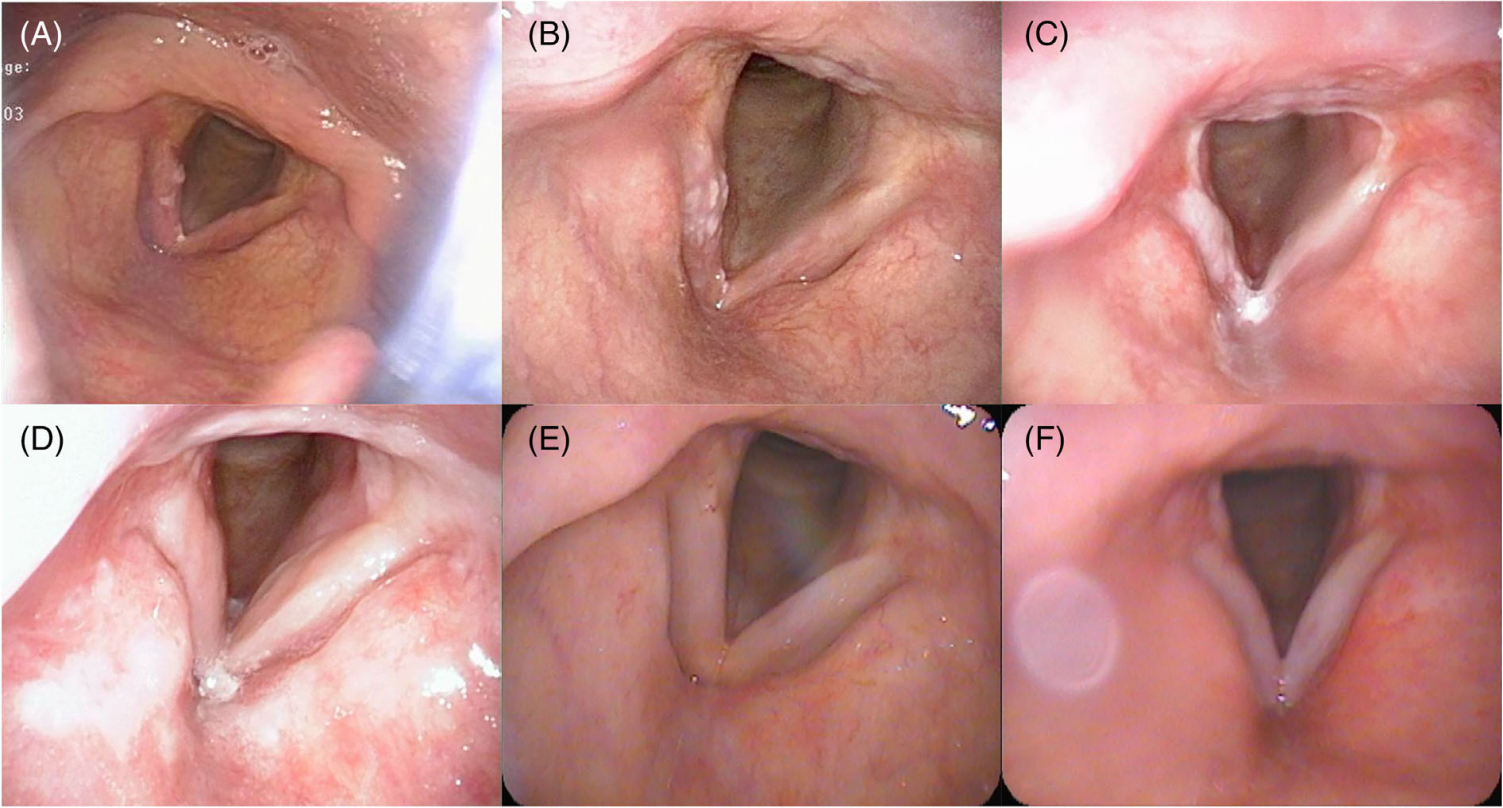
Laryngoscopy findings of the pharynx before treatment and during the follow‐up period. (A) Pre‐treatment. (B) At 3 days after radiotherapy (RT) initiation. (C) At 2 weeks after RT initiation. The patient had resumed smoking, and laryngoscopy findings revealed a slightly changed bilateral vocal cord mucosa with a white lesion. (D) Completion of RT. (E) At 1 year after RT. (F) At 13 months after RT. The patient resumed smoking one pack per day of cigarettes

**FIGURE 2:**
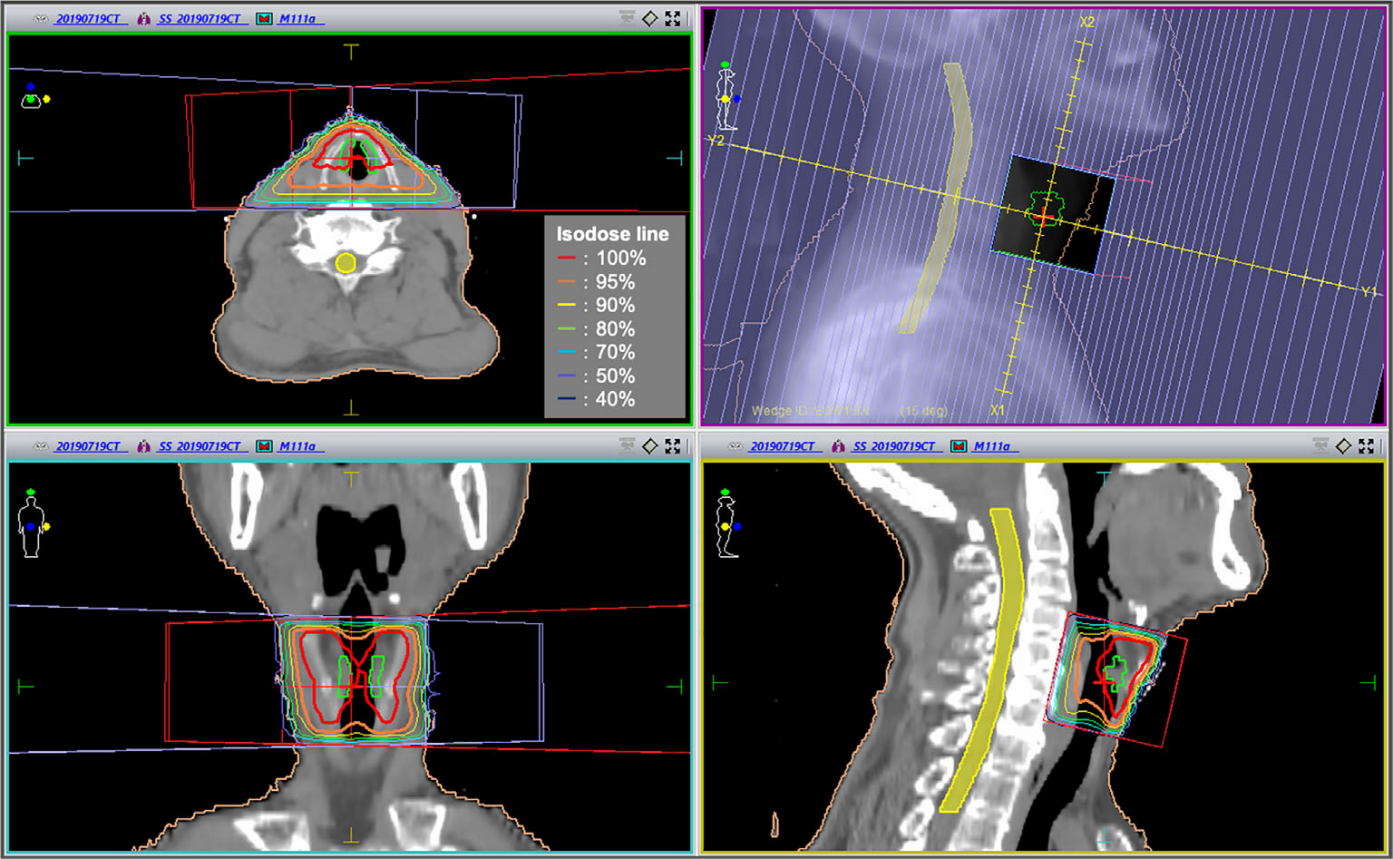
Planning CT image of RT. The RT technique was a three‐dimensional conventional technique using a dynamic wedge. The radiation field size was 5.5 × 6 cm with a conventional rectangle field (no multi‐leaf collimator). CT, computed tomography; RT, radiotherapy

A 63‐year‐old man presented with hoarseness that continued for 6 months. Laryngoscopy revealed a white lesion in the right vocal cord (Figure 1A). Biopsy of the right vocal cord was performed, and the patient was diagnosed with glottic squamous cell carcinoma. The patient reported drinking 1500 ml beer daily and had smoked one and a half packs of cigarettes per day for over 40 years. He quit smoking at the time of diagnosis. Computed tomography (CT) findings revealed no lymph node or distant metastasis. The patient's clinical stage was T1aN0M0, I (TNM classification, 8th edition). Definitive RT alone was planned. The RT dose was 63 Gy delivered in 28 fractions for the larynx using a three‐dimensional conventional RT technique with a dynamic wedge. The radiation field size was 5.5 × 6 cm with a conventional rectangular field (no multi‐leaf collimator) (Figure 2). At 3 days after RT initiation, laryngoscopy revealed that the primary tumor of the right vocal cord had slightly increased in size (Figure 1B). At 2 weeks after RT initiation, the patient had resumed smoking, and laryngoscopy findings revealed a slightly changed bilateral vocal cord mucosa with a white lesion (Figure 1C). He was instructed to quit smoking again. However, he continued drinking 1500 ml beer daily during RT. RT was completed on schedule with no pauses (total treatment time was 42 days). At the time of RT completion, laryngoscopy revealed that the primary tumor had disappeared (Figure 1D). Acute toxicities included grade 2 dermatitis and grade 1 mucositis. The initial treatment response was complete response (CR), and the follow‐up period was started.

**FIGURE 3:**
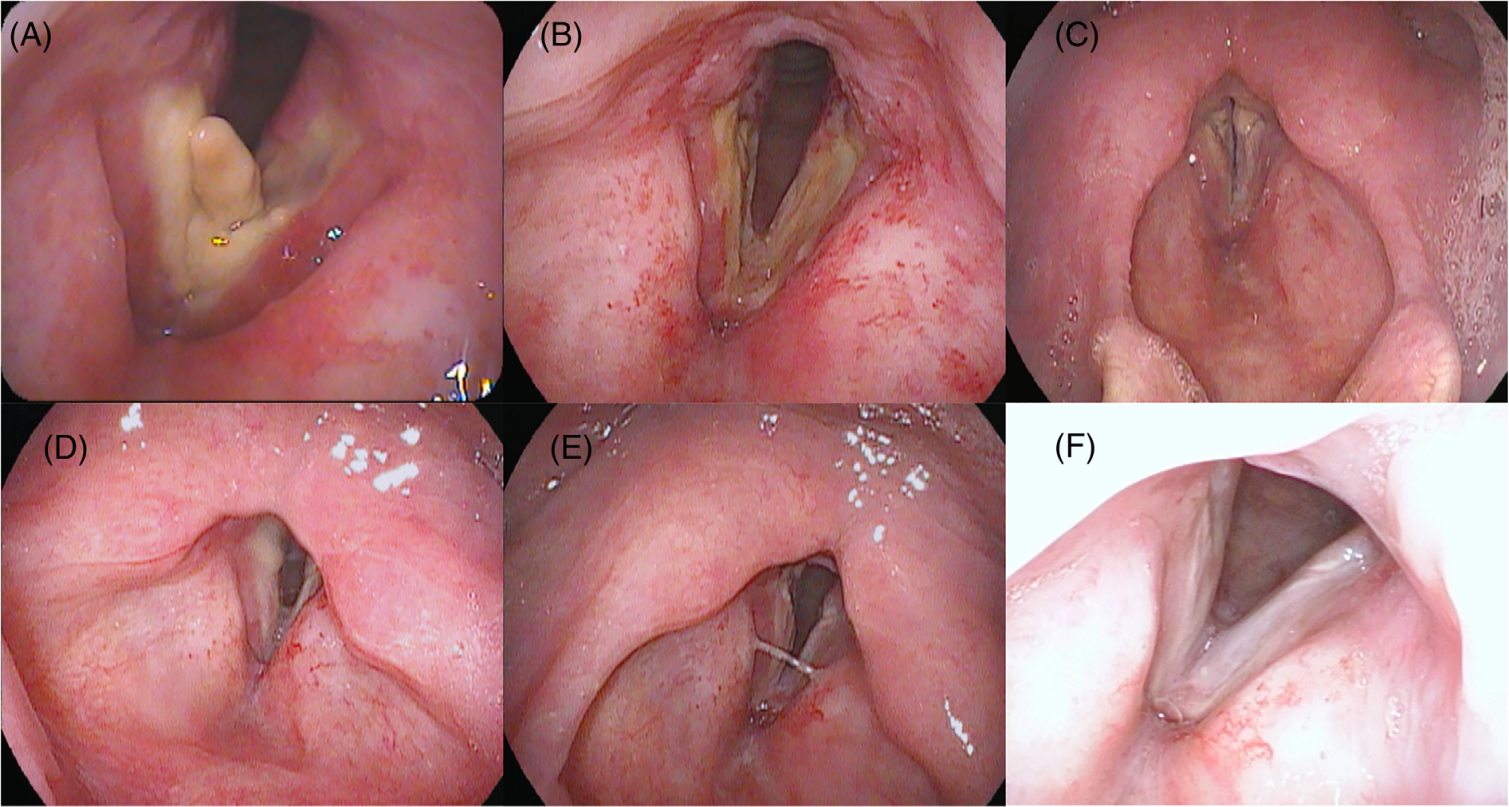
Laryngoscopy findings of the pharynx during the follow‐up period. (A) At 15 months after radiotherapy, a large tumor in the larynx was observed. (B) The day of surgical excision of the laryngeal tumor. (C) At 3 weeks postoperatively. The patient suddenly had dyspnea, and laryngoscopy revealed total laryngeal paralysis. (D) At 6 days, (E) 12 days, and (F) 1 month after transvenous steroid administration

**FIGURE 4:**
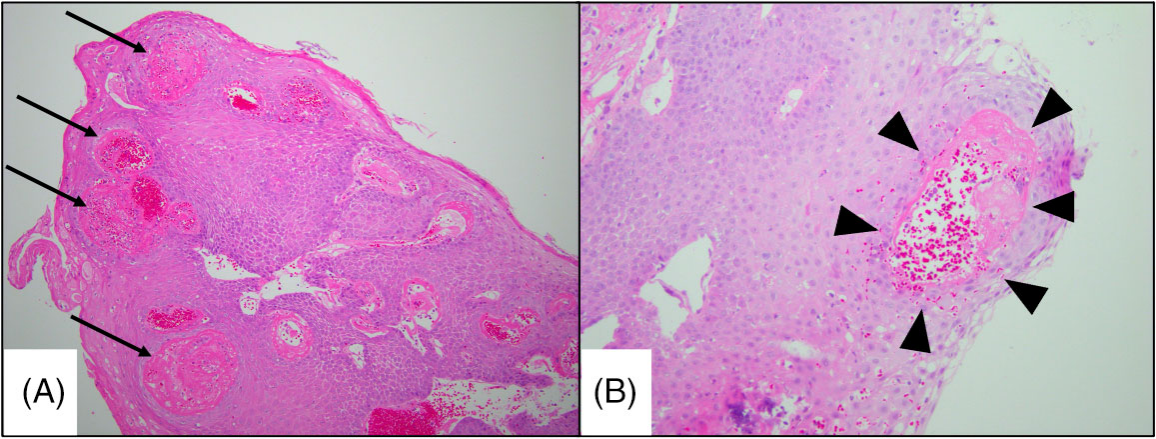
Hematoxylin–eosin‐stained sections (A) at ×100 magnification and (B) at ×200 magnification of the pharyngeal tumor at 13 months after radiotherapy. Significant inflammatory cell infiltration is observed. There was no recurrence of the primary cancer. Significant vitrification of blood vessel walls (arrow) and narrowing of the vascular lumen (arrow head) were observed. These typical findings presented ischemic change of the mucosa caused by late radiation toxicity

**FIGURE 5:**
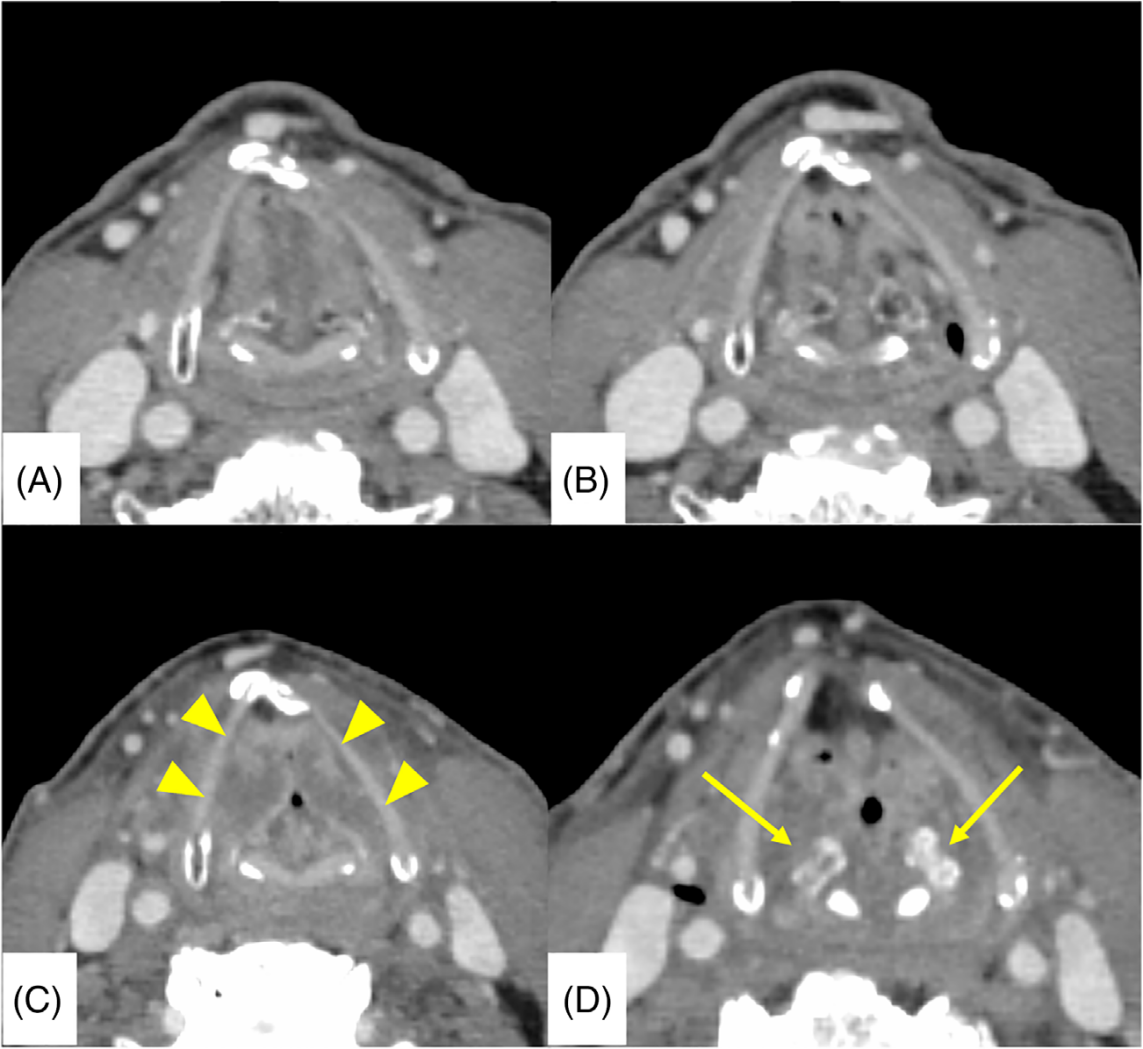
Comparison of computed tomography (CT) axial images obtained before treatment (A, B) and at the time of total laryngeal paralysis (C, D). Post‐treatment CT reveals severe edema in the bilateral vocal cord (arrow head). Bilateral arytenoid cartilage shows atrophy and sclerosis (arrow). There was no recurrence of the primary cancer. These findings indicated late radiation toxicity

Monthly examination was performed, and no local recurrence or metastasis was observed at 1 year after RT (Figure 1E). During the follow‐up period, although the patient had quit smoking, he continued to drink 1500 mL beer daily. At 13 months after RT, he resumed smoking one pack per day of cigarettes. At 1 month after resuming smoking, laryngoscopy revealed a slightly changed bilateral vocal cord with a white lesion without recurrence (Figure 1F). At 2 months after resuming smoking, he had severe sore throat and hoarseness. Laryngoscopy findings revealed a large tumor in the larynx (Figure 3A). Unfortunately, we did not perform CT at that time. Eleven days later, surgical excision of the tumor was performed (Figure 3B), and the patient was histologically diagnosed with radiation‐induced laryngeal necrosis as a late toxicity without cancer recurrence (Figure 4). After surgical excision, the patient continuously experienced sore throat despite oral painkiller administration. At 3 weeks postoperatively, he suddenly developed dyspnea, and laryngoscopy findings revealed total laryngeal paralysis (Figure 3C). The patient underwent an emergent tracheostomy. After tracheostomy, the lingering sore throat disappeared momentarily. Enhanced CT revealed bilateral vocal cord edema, and bilateral arytenoid cartilage showed atrophy and sclerosis (Figure 5). No cancer recurrence was observed on enhanced CT findings. Transvenous administration of steroids alone was performed. First, we injected hydrocortisone 500 mg per day for 2 days, with a gradually decreasing dose (300 mg per day × 2 days followed by 200 mg per day × 2 days). Laryngoscopy findings revealed a slight improvement in vocal cord movement and necrosis (Figure 3D). Thus, we switched to oral prednisolone. We administered prednisolone 30 mg per day for 2 days, with a gradually decreasing dose (20 mg per day × 2 days followed by 10 mg per day × 2 days; then, 5 mg per day × 2 days). Steroid treatment further improved the laryngeal necrosis and laryngeal paralysis (Figure 3E). After oral administration of prednisolone, the patient was discharged with tracheostomy. At 2 weeks after discharge (1 month after tracheostomy), laryngoscopy findings revealed that the bilateral vocal cord further improved the movement and necrosis similar to the pre‐tracheostomy status (Figure 3F). No respiratory distress was observed even when the tracheal foramen was blocked.

## 3 DISCUSSION

RT plays an important role in the definitive treatment of glottic cancers with a high local control rate and few severe toxicities. The National Comprehensive Cancer Network guidelines recommend a dose of 63 (2.25 Gy/fraction, preferred) to 66 Gy (2 Gy/fraction) for T1N0 glottic cancers.^7^ Chera et al. retrospectively reviewed the data of 585 patients with T1N0 to T2N0 glottic squamous cell carcinoma treated with RT alone at the University of Florida.^1^ In their study, in 253 patients with T1a glottic cancer, the 5‐ and 10‐year local control rates were 94 and 93%, respectively. In our case, we also achieved CR for primary tumors treated with RT alone and delivered 63 Gy in 28 fractions.

Although there are few severe complications of RT for early glottic cancer, RLN is the most severe late toxicity of RT. In the follow‐up period after the initial treatment, clinical oncologists occasionally experience difficulty in diagnosing true cancer recurrence or RLN. Thus, most RLN cases had been diagnosed by total laryngectomy without histological recurrence of carcinoma.^1^, ^8^ In our case, we suddenly observed a large tumor in the larynx by laryngoscopy at 15 months after RT (Figure 3A), despite no abnormal lesion in the larynx was observed at 13 months after RT (Figure 1F). Moreover, the laryngeal tumor showed a white and necrotic appearance. Therefore, we considered that the tumor was an inflammatory reaction induced by resumption of smoking without cancer recurrence. The pathological diagnosis also supported the presence of RLN. A CT scan of the total laryngeal paralysis revealed severe edema of the bilateral vocal cord, and bilateral arytenoid cartilage showed atrophy and sclerosis (Figure 5). These findings also indicated late radiation toxicity. Keene et al, in their histopathological study, reported the presence of radionecrosis in laryngeal carcinoma cases,^8^ and documented that the arytenoid cartilage was most frequently involved when chondronecrosis occurred in association with RT. Fortunately, steroid administration was significantly effective for RLN in our case and, therefore, we could avoid total laryngectomy. RLN was only localized to the mucosa, which may have been associated with the effect of steroids. However, further follow‐up examinations are needed to determine whether RLN worsens. In this case, the patient continued to have sore throat 3 weeks after surgical excision of the laryngeal tumor and 15 months after RT; finally, he developed total laryngeal paralysis. Therefore, when the sore throat continues post‐surgical resection of necrotic tissue of the larynx, attention must be paid to reduce the risk of total laryngeal paralysis.

Smoking influences hemoglobin by the binding of carbon monoxide (CO) to hemoglobin, leading to the formation of carboxyhemoglobin (HbCO).^9^ HbCO causes tissue hypoxia because of the decreased hemoglobin levels. High HbCO levels can lead to tissue hypoxia and result in radiation resistance of the tumor in mouse models.^10^ Hoff et al's prospective study reported the effect of smoking on outcomes in patients treated with RT for head and neck squamous cell carcinoma.^11^ In their study, heavy smokers (>1 pack/day) had significantly poorer outcomes of 5‐year locoregional control (44 vs. 65%, *p* = .001), disease‐specific (56 vs. 77%, *p* = .003), and overall survival (39 vs. 66%, *p* = .0004) compared to non‐smokers. Chen et al's prospective study demonstrated smoking cessation during curative chemoradiotherapy for 63 patients with head and neck squamous cell carcinoma.^12^ Forty‐one patients (65%) successfully discontinued smoking throughout chemoradiotherapy (CRT). During a median 33‐month follow‐up period, the patients with successful smoking cessation during CRT presented significantly fewer acute toxicities (*p* = .01) and reduced tumor progression risks (hazard ratio: 0.4, *p* = .05).

Therefore, interventions for smoking cessation are important for patients with head and neck cancers. However, despite the well‐known adverse effects of smoking, complete smoking cessation is difficult in patients with head and neck cancers. Conlon et al conducted a prospective study assessing cigarette smoking characteristics and interest in cessation in patients with head and neck cancers using a questionnaire.^13^ In the analysis of 183 current smoker patients at the time of enrollment, most had previously attempted to quit smoking (77.0%), and 58.5% of the current smokers had resumed smoking within 1 year. Similar to that report, in our case, although the physician in charge warned the patient to quit smoking several times, the patient resumed smoking during and after RT. Considering the laryngoscopy findings during the follow‐up period and the resumption of smoking, we hypothesized that smoking induced RLN. This case suggested that smoking has a greater risk of cancer recurrence and RLN development in patients with glottic cancer who receive definitive RT. Therefore, medical oncologists should approach patients' smoking cessation more actively.

## 4 CONCLUSIONS

We report a case of smoking‐induced RLN for T1a glottic squamous cell carcinoma treated with RT alone. This rare case suggests that smoking has a greater risk of cancer recurrence and RLN development in patients with glottic cancer who receive definitive RT. Rather than leaving smoking cessation up to the patient, it would be necessary for clinicians to actively intervene to help patients continue their effort to quit smoking.

## CONFLICT OF INTEREST

The authors declare there is no conflict of interest.

## AUTHOR CONTRIBUTIONS

YT: Conceptualization; data curation; formal analysis; investigation; methodology; project administration; resources; supervision; validation; visualization; writing ‐ original draft; writing‐review & editing. SI: Writing‐review & editing. MA; Writing‐review & editing. YU; Writing‐review & editing. KO; Writing‐review & editing. JK; Data curation; investigation; methodology; project administration; visualization; writing‐review & editing. MN; Writing‐review & editing. YK; Data curation; investigation; methodology; visualization; writing‐review & editing. SI; Writing‐review & editing. MK; Writing‐review & editing.

## ETHICS STATEMENT

Written informed consent was obtained from the patient for publication of this case report and accompanying images.

## Data Availability

The data that support the findings of this study are available from the corresponding author upon reasonable request.
